# Hospitalized children with SARS-CoV-2 infection and MIS-C in Jamaica: A dive into the first 15 months of the novel pandemic

**DOI:** 10.3389/fped.2022.904788

**Published:** 2022-09-07

**Authors:** Crista-Lee Shahine Berry, Roxanne Helene Melbourne-Chambers, Abigail Natalie Harrison, Joshua James Anzinger, Kelly-Ann Maxorinthia Gordon-Johnson, Varough Mohamed Deyde, Celia Dana Claire Christie

**Affiliations:** ^1^Department of Child and Adolescent Health, University of the West Indies, Kingston, Jamaica; ^2^Department of Child and Adolescent Health (Neurology), University Hospital of the West Indies, Kingston, Jamaica; ^3^Department of Child and Adolescent Health (Adolescent Health), University Hospital of the West Indies, Kingston, Jamaica; ^4^Department of Microbiology (Virology), University of the West Indies, Kingston, Jamaica; ^5^Global Virus Network, Baltimore, MD, United States; ^6^Centers for Diseases Control and Prevention, Caribbean Regional Office, Kingston, Jamaica; ^7^Department of Child and Adolescent Health (Infectious Diseases), University Hospital of the West Indies, Kingston, Jamaica

**Keywords:** COVID-19 outcomes in Jamaican Children, COVID-19, children, adolescents, multisystem inflammatory syndrome of children, MISC, Jamaica, Caribbean

## Abstract

**Objectives:**

COVID-19 in children was initially mild until the emergence of Multisystem Inflammatory Syndrome in Children (MIS-C). We describe pediatric COVID-19 in a developing country within the Caribbean.

**Methods:**

Jamaican children who were hospitalized with SARS-CoV-2 infection, in one Caribbean regional academic referral center from April 2020 through June 2021 were included. Prospective surveillance and pediatric infectious disease consultations were performed using the CDC's MIS-C case definition. Data were extracted from patients' hospital charts using WHO's reporting form, entered into the RedCap database, and SPSS 28 was used for analysis. MIS-C and non-MIS-C patients were compared using independent sample *t*-tests for continuous variables and Fisher's exact test for categorical variables, *p* values < 0.05 were statistically significant.

**Results:**

Seventy-nine children with COVID-19 with/without MIS-C presented to UHWI. Thirty-eight (48%) were mild ambulatory cases. Hospitalizations occurred in 41 (52%) children, with median age of 10 $\frac{1}{2}$ years. SARS-CoV-2 RT-PCR positivity was present in 26 (63%), Immunoglobulin M, or Immunoglobulin G (IgM/IgG) positivity in 8 (20%), with community exposures in 7 (17%). Eighteen (44%) MIS-C positive patients were significantly more likely than 23 MIS-C negative patients (56%) to present with fever (94% vs. 30%; *p* < 0.001), fatigue/lethargy (41% vs. 4%; *p* = 0.006), lymphadenopathy (33% vs. 0%; *p* = 0.003), elevated neutrophils (100% vs. 87%; *p* = 0.024), and ESR (78% vs. 9%; *p* = 0.002). Involvement of > two organ systems occurred more frequently in MIS-C positive cases (100% vs. 34%; *p* < 0.001), including gastrointestinal (72% vs. 17%; *p* < 0.001); vomiting/nausea (39% vs. 9%; *p* < 0.028); hematological/coagulopathic (67% vs. 4%; *p* < 0.001); dermatologic involvement (56% vs. 0%; *p* < 0.001); and mucositis (28% vs. 0%; *p* = 0.001). MIS-C patients had Kawasaki syndrome (44%), cardiac involvement (17%), and pleural effusions (17%). MIS-C patients had >4 abnormal inflammatory biomarkers including D-dimers, C-reactive protein, ESR, ferritin, troponins, lactate dehydrogenase, neutrophils, platelets, lymphocytes, and albumen (72%). MIS-C patients were treated with intravenous immune gamma globulin (78%), aspirin (68%), steroids (50%), and non-invasive ventilation (11%). None required inotropes/vasopressors. MIS-C negative patients received standard care. All recovered except one child who was receiving renal replacement therapy and developed myocardial complications.

**Conclusions:**

In this first report of COVID-19 from the Caribbean, children and adolescents with and without MIS-C were not very severe. Critical care interventions were minimal and outcomes were excellent.

## Background

There were no reported cases of the novel coronavirus in children aged less than 18 years until ten weeks into the epidemic in China (1). Subsequently, child and adolescent cases were reported from China, Italy, Iceland, and the United States (1–4). Initially, COVID-19 in children was either asymptomatic or had a milder course with a better prognosis than in adults (5). By April 2020, the post-infectious multisystem inflammatory syndrome in children (MIS-C), the Pediatric Inflammatory Multisystem Syndrome (PIMS-Ts), was being recognized in children with COVID-19 (6–10). Case definitions for MIS-C/PIMS-Ts were first created by the USA's Centers for Disease Control and Prevention (CDC), the World Health Organization (WHO), and Royal College of Pediatrics and Child Health (RCPCH) (6–10). The American Academy of Pediatrics and American College of Rheumatology later recommended a treatment approach for children with MIS-C (11, 12).

The pediatric COVID-19 pandemic has since expanded globally (13–17). The severity of illness and outcomes in children vary based on demographic factors, circulating SARS-CoV-2 variants of concern, patient age, comorbid illnesses, a diagnosis of MIS-C, and timely access to optimal medical care (13–19). In the USA, during the SARS-CoV-2 Omicron outbreak, children aged <4 years were being hospitalized at > 5 times the observed rate during the peak of the Delta variant predominance (19). As in adults, children and adolescents are also experiencing the effects of “Long COVID-19” (18). Globally, access to COVID-19 vaccination in adolescents has lagged behind adult immunizations and is disproportionate in developing as compared to developed countries (20). Also, COVID-19 vaccines are not routinely recommended for children without comorbidities, and vaccine hesitancy is accentuated by increased reports of adverse events of myocarditis and pericarditis in youth (20). Indirect adverse effects of the global pandemic on children and adolescents have included mental health concerns, school closures, loss of educational opportunities, missed vaccinations, and death of their caregivers (21).

The Caribbean has a population of about 44.2 million residents in 700 islands, comprising 44 countries, or territories that are bordered by the land masses of North, Central, and South America and the waters of the Caribbean Sea, the Gulf of Mexico, and the Atlantic Ocean (22). The Caribbean is diverse in its ethnicity, culture, socioeconomic status, and language and is highly dependent on tourism to sustain its economic viability (22). Jamaica, with a Gross National Income (GNI) per capita of USD 4,990 (2018, World Bank) is an upper middle-income, developing, Caribbean Island nation of 2.9 million inhabitants, which diagnosed its first imported case of COVID-19 on March 10, 2020, one day before COVID-19 was declared a pandemic by the WHO (22, 23). “Face-to-face” school attendance was suspended, and virtual classes began approximately five weeks later. Varied public health measures were implemented. Community transmission was declared in September 2020 with test positivity rates fluctuating between 8 and 41%, peaking in March 2021. By June 1st, 2021, Jamaica reported 48,639 COVID-19 confirmed cases, of these 10.3% (4999) were aged <18 years. Of these 4,999 confirmed cases, five percent (250) were under one year old, 10.4% (520) were aged 1–2 years, 13.1% (653) 3–5 years, 36.1% (1,807) 6–12 years, and 35.4% (1769) 13–17 years old. Deaths were reported in 0.1% and 0.2% in those aged <10 years and 10–19 years old, respectively (23). These national data were gleaned from the Jamaican Ministry of Health and Wellness for patients presenting for treatment and care in hospitals and primary care settings. The Caribbean Public Health Agency reported the B.1.1.7 (Alpha) SARS-CoV-2 as the only “variant of concern” identified in Jamaica through the third week of June 2021 (24). According to sequencing data that were reported by the United States' Centers for Disease Control and Prevention, among samples that were selected by Jamaica's National Public Health Laboratory, the SARS-CoV-2 “Delta” variant was first seen in a sample that was collected on 11 February, 2021 (gisaid.org) and reported 23 June, 2021 (25). While “Omicron” and its sub-variants were first seen 21 December, 2021 (gisaid.org) and they have been circulating in Jamaica since then (26).

The University Hospital of the West Indies (UHWI), Jamaica, is a tertiary academic institution that accepts referred and ambulatory adult and pediatric patients island-wide and throughout the Caribbean for hospitalization including intensive care. The UHWI has approximately 120 inpatient beds for patients aged 0–16 years. SARS-CoV-2 RT-PCR nasopharyngeal testing is performed on every hospitalized patient. SARS-CoV-2 antibody testing is done when indicated, especially in patients suspected with MIS-C, who test negative for SARS-CoV-2. Although SARS-CoV-2 RT-PCR has very high analytical sensitivity, diagnostic sensitivity is imperfect and false negatives range from 1.8 to 58% (27). The use of SARS-CoV-2 RT-PCR followed by antibody testing in those with a presumed diagnosis of MIS-C increases the diagnostic sensitivity for this disease, which usually occurs up to 6 weeks or more after the virus is cleared from the upper respiratory tract (28).

This report documents COVID-19 in children presenting during the first 15 months of the global pandemic in a resource-constrained single-center teaching hospital in Jamaica and is the first of its kind from a developing country within the Caribbean region. It provides historical context and also informs clinicians in similar settings as the pandemic rapidly evolves. We describe the clinical characteristics, investigations, therapy, and outcomes in all children aged <16 years old who were hospitalized at UHWI with acute SARS-CoV-2 infection, and/or MIS-C from March 2020 through June 2021 of the global COVID-19 pandemic. The clinical characteristics and outcomes between patients presenting with MIS-C and those without MIS-C were compared.

## Methods

This descriptive ambispective cohort study included all admitted patients, aged <16 years, with SARS-CoV-2 infection presenting to the UHWI from all over the island from April 1, 2020, through June 30, 2021. Patients who were hospitalized at the UHWI, Jamaica, who met the CDC's case definition for MIS-C and non-MIS-C patients were further analyzed in detail for this report (9). The MIS-C diagnostic criteria included fever for >24 h, laboratory evidence of inflammation with at least one of the following criteria: elevated C-reactive protein, erythrocyte sedimentation rate (ESR), fibrinogen, procalcitonin, D-dimer, ferritin, lactic acid dehydrogenase, interleukin-6, elevated neutrophils, or reduced lymphocytes and or albumin and clinically severe illness requiring hospitalization, with multisystem (>2) organ involvement, including cardiac, renal, respiratory, hematologic, gastrointestinal, dermatologic, or neurological; no alternative diagnoses; evidence of current, or recent SARS-CoV-2 infection by RT-PCR, serology, or antigen test; or exposure to a suspected, or confirmed COVID-19 case in the 4 weeks prior (9). Organ system involvement was assessed with supporting clinical features and laboratory and other related investigations. Children meeting full, or partial criteria for Kawasaki disease, or a pediatric death from SARS-CoV-2 infection are also considered MIS-C (9).

Children were managed using treatment guidelines as they evolved (8, 12). Children with MIS-C received intravenous gamma globulin (2 g/kg as a single infusion over 10–12 h), steroids (methylprednisolone intravenously 2 mg/kg/day in two divided doses, transitioned to oral prednisolone, or prednisone which is tapered over two to four weeks), acetyl salicylic acid [aspirin] (80–100 mg/kg per day divided into four doses), and anticoagulants (such as enoxaparin). Other supporting modalities included blood and blood products, antibiotics, fluids, oxygen, and other treatment modalities, as appropriate. Those without MIS-C received standard care, which included investigations of complete blood count, electrolytes, renal function, blood and urine culture, antipyretics, fluids, oxygen, dexamethazone, anti-thrombotic agents and antibiotics, if needed. All patients were treated for their underlying comorbidities, as deemed appropriate.

Hospitalized patients have mandatory nasopharyngeal RT-PCR testing for SARS-CoV-2.

All suspected and confirmed hospitalized COVID-19 patients had written Pediatric Infectious Diseases consultation(s) in accordance with UHWI's COVID-19 hospital policy (all on file). Upon hospital discharge, the patients were reviewed and followed by the pediatric inpatient or outpatient department. SARS-CoV-2 infection was confirmed by real-time RT-PCR positivity of nasopharyngeal samples at the National Influenza Center, which is housed at the UHWI's Diagnostic Virology Laboratory, the presence of IgG and/or IgM antibodies (Abbott Architect), and/or a confirmed epidemiological link, or presumed contact with an infected person during the community outbreak. SARS-CoV-2 antibody testing was performed in those with suspected MIS-C but with negative RT-PCR tests. Electronic medical and laboratory records and paper clinical records were reviewed by senior researchers and a clinical research assistant. Results of clinical features, investigations, treatments, and outcomes were extracted from patients' hospital charts using a modified WHO case report form, and data were entered into RedCap database by senior clinical researchers.

Statistical analyses were performed using IBM SPSS version 28 [International Business Machines Corporation (IBM), Armonk, New York, USA]. Descriptive statistics were obtained for clinical characteristics and outcomes in children aged <16 years old on admission using mean and standard deviation for continuous variables and number and percent for categorical variables. Comparison of differences between children with or without MIS-C was computed using independent samples *t*-tests for continuous variables and Fisher's exact test for categorical variables. A *p*-value < 0.05 was considered statistically significant.

## Results

During April 2020 to June 2021, 79 children and adolescents aged <16 years presented through the UHWI's Emergency Room with SARS-CoV-2 RT-PCR positive status, or COVID-19, 41 (52%) were hospitalized and 18 (44%) of these met the CDC's case definition for MIS-C.

Among 41 (52%) patients who were hospitalized, the median age was 10.5 years (range 0–16 years); SARS-CoV-2 RT-PCR positivity was present in 26 (63.4%) and SARS-CoV-2 IgM/IgG positivity in 8 (20%), the rest 7 (17%) had community exposures (Table 1). Twenty-one (51.2%) were males and 40 (98%) were of mixed Afro-Caribbean ethnicity. Twenty-two (53.7%) had comorbidities; of these, Sickle cell disease (27.3%), asthma (27.3%), and immunodeficiency (18.2%) were the most common. The prevalence of comorbidities was similar in patients with MIS-C and without MIS-C (Table 1).

**Table 1:** Characteristics of children hospitalized with COVID-19, MIS-C^*^ vs non-MIS-C.

	**Total cases (%) *N* = 41**	**MIS-C cases (%) *N* = 18**	**Non-MIS-C cases (%) *N* = 23**	***p*-Value**
Male	21 (51.2%)	9 (50%)	12 (52%)	1
**Confirmation**				<0.001
SARS-CoV-2 RT PCR+	26 (63.4%)	4 (22.2%)	22 (95.7%)	
SARS-CoV-2 IgG/IgM+	8 (19.5%)	8 (44.4%)	0 (0%)	
No confirmatory test	7 (17%)	6 (33.3%)	1 (4.3%)	
**Age Groups**				0.041
Infant (<1 year)	8 (19.5%)	1 (5.6%)	7 (30.4%)	
Toddler (1–2 years)	6 (14.6%)	4 (22.2%)	2 (8.7%)	
Pre-schooler (3–5 years)	2 (4.9%)	2 (11.1%)	0 (0%)	
Middle Childhood (6–11 years)	9 (22%)	6 (33.3%)	3 (13.0%)	
Adolescent (12–16 years)***	16 (39%)	5 (27.8%)	11 (47.8%)	
**Comorbidities**	22 (53.7%)	9 (50%)	13 (56.5%)	0.758
Congenital Heart Disease	1 (4.5%)	0 (0%)	1 (7.7%)	1
Hypertension	4 (18.2%)	2 (22.2%)	2 (15.4%)	1
Asthma	6 (27.3%)	3 (33.3%)	3 (23.1%)	0.655
Chronic Kidney Disease	2 (9.1%)	1 (11.1%)	1 (7.7%)	1
Chronic Lung Disease (not asthma)	1 (4.5%)	0 (0%)	1 (7.7%)	1
Obesity	3 (13.6%)	2 (22.2%)	1 (7.7%)	0.544
Epilepsy	1 (4.5%)	0 (0%)	1 (7.7%)	1
Immunosuppression, Malignancy	4 (18.2%)	1 (11.1%)	3 (23.1%)	0.616
Sickle Cell Disease, SCD	6 (27.3%)	4 (44.4%)	2 (15.4%)	0.178
Other Comorbidities (Autism Spectrum D/O, Dysmorphic Syndrome, Hirschsprung's Disease, Metabolic Bone Disease, Sickle Nephropathy, Steroid Induced Diabetes Mellitus)	8 (36.4%)	3 (33.3%)	5 (38.5%)	1

*Multisystem Inflammatory Syndrome in Children (MIS-C) with COVID-19, MIS-C.

** RT-PCR was performed on ALL hospitalized patients. Antibody testing was not performed if the RT-PCR test was positive. Hence, no patient in the non-MIS-C group had antibody tests performed.

***Children aged <16 years are all admitted to the pediatric floors, those aged 16 years and older on presentation, are hospitalized on the adult floors. However, one child celebrated his 16th birthday during his pediatric hospitalization.

The denominators may vary (eg., co-morbidities).

Twenty-two of the 23 non-MIS-C patients (95.7%) were RT-PCR positive, 11 (47.8%) were asymptomatic, 12 (52.2%) had mild symptoms, including fever (30.4%), cough (18.2%), and abdominal pain (21.7%) (Tables 1, 2A). Sickle cell disease and asthma were the most common comorbidities. In symptomatic acute cases, it was clinically impossible to ascribe the cough and respiratory distress to these diseases or to the acute SARS-CoV-2 infection. Similarly, in those with acute appendicitis, it was challenging to ascribe the abdominal pain to appendicitis or acute COVID-19. Reasons for hospitalization included bony fracture, hematocolpus from an imperforate hymen, Sickle cell disease, chronic kidney disease for hemodialysis, malignancy, appendicitis, diabetic ketoacidosis, and four well neonates whose mothers were RT-PCR positive within 48 h of delivery.

**Table 2A:** Clinical symptoms of children hospitalized with COVID-19, MIS-C^*^ vs. non-MIS-C.

Symptoms**	**Total cases (%) *N* = 41**	**MIS-C cases (%) *N* = 18**	**Non-MIS-C cases (%) *N* = 23**	***p*-Value**
Fever	24 (58.5%)	17 (94.4%)	7 (30.4%)	<0.001
Cough	8 (20 %)	4 (22.2%)	4 (18.2%)	1
Sore throat	7 (18.4%)	5 (29.4%)	2 (9.5%)	0.207
Rhinorrhoea	6 (14.6%)	5 (27.8%)	1 (4.3%)	0.07
Shortness of Breath	7 (17.1%)	4 (22.2%)	3 (13%)	0.679
Chest Pain	3 (8.1%)	1 (5.9%)	2 (10%)	1
Myalgia	4 (11.1%)	3 (18.8%)	1 (5%)	0.303
Joint Pains	4 (10.8%)	3 (17.6%)	1 (5%)	0.315
Fatigue/Lethargy	8 (20%)	7 (41.2%)	1 (4.3%)	0.006
Headache	4 (10.8%)	3 (17.6%)	1 (5%)	0.315
Seizures	1 (2.4%)	0 (0%)	1 (4.3%)	1
Abdominal Pain/tenderness	13 (31.7%)	8 (44.4%)	5 (21.7%)	0.179
Vomiting/Nausea	9 (22%)	7 (38.9%)	2 (8.7%)	0.028
Diarrhea	5 (12.2%)	4 (22.2%)	1 (4.3%)	0.15
Conjunctivitis	6 (14.6%)	4 (22.2%)	2 (8.7%)	0.377
Skin Rash	9 (22%)	9 (50%)	0 (0%)	<0.001
Lymphadenopathy	6 (14.6%)	6 (33.3%)	0 (0%)	0.004
Bleeding/Bruising	4 (9.8%)	3 (16.7%)	1 (4.3%)	0.303
Mucositis	5 (12.2%)	5 (27.8%)	0 (0%)	0.011
Other–signs/symptoms Anorexia, Arthritis, Irritability, Nasal congestion, Odynophasia, Pedal edema, Scrotal pain/tenderness	15 (36.6%)	11 (61.1%)	4 (17.4%)	0.008

*Multisystem Inflammatory Syndrome in Children with COVID-19, MIS-C.

**Symptoms with frequency <1 (<4.3), for both groups, were omitted.

The denominators may vary.

Eighteen (44%) hospitalized patients were diagnosed with MIS-C: 4 (22%) were SARS-CoV-2 RT-PCR positive, 8 (44%) were SARS-CoV-2 IgM/IgG positive, and 6 (33%) had a presumed household or community contact to someone with SARS-CoV-2 infection (Table 1). Seventeen MIS-C patients had a history of fever, or documented fever, while the one remaining patient met the MIS-C criteria due to demise with COVID-19 (9). The 18 MIS-C patients compared to 23 non-MIS-C patients presented with documented fever in hospital for >24 h (94% vs. 30%, *p* < 0.001), fatigue/lethargy (41% vs. 4%; *p* = 0.006), and lymphadenopathy (33% vs. 0%; *p* = 0.004). Other signs and symptoms are shown (Table 2A). All MIS-C patients had >2 organ systems involved (100% vs. 34.8%, *p* < 0.001) (Table 2B); with gastrointestinal symptoms (72% vs. 17%; *p* < 0.001) including/nausea (39% vs. 9%; *p* = 0.028), dermatological symptoms (56% vs. 0%; *p* < 0.001) with mucositis (28% vs. 0%; *p* = 0.011); neurological 33.3%, hematological/coagulopathic (67% vs. 4%; *p* < 0.001), respiratory 50% with pleural effusions (17% vs. 0%; *p* = 0.077) and cardiac symptoms (17% vs. 0%; *p* = 0.077) (Tables 2A, B), with elevated troponin-T (17% MIS-C), myocardial dysfunction (5.6%), and coronary arteritis (5.6%) (Tables 3, 4). Of the 18 MIS-C patients, 44% (8) fulfilled the criteria for Kawasaki disease; one was SARS-CoV-2 RT PCR positive, four were SARS-CoV-2 IgG/IgM positive, and the remaining three were serologically negative with a history of community or household contact. There was the one sudden death (5.6%) in a child who was SARS-CoV-2 RT-PCR positive with a comorbidity.

**Table 2B:** Description of mucocutaneous manifestations in the hospitalized children and adolescents with MIS-C.

**Case Numbers**	Dermatological/Mucocutaneous Manifestation of Multisystem Inflammatory Syndrome, MIS-C*
1	Generalized maculo-papular pruritic rash.
2	Fine erythematous papules to limbs, Strawberry tongue. Redness to palms and soles
3	Pruritic, erythematous maculo-papular lesions to upper and lower extremities, including the palms and soles
4	Erythematous patches and plaques on face, torso, arms, and feet
5	Fine erythematous pruritic papules. Edema and erythema to palms and soles. Desquamation to perianal region. Redness and sores intra-oral and strawberry tongue
6	Small ulcers in mouth and tongue. Vesicles to upper lip
7	Small non-pruritic, non-erythematous papules to the forehead and chin. Strawberry tongue.
8	Dry cracked lips
9	Generalized fine papular pruritic rash to body, sparing the face, palms, and soles. Sandpaper-like consistency.
10	Multiple erythematous painful erythematous urticarial-like nodules to face, neck, chest, abdomen, back, and arms.

*Multisystem Inflammatory Syndrome in Children with COVID-19, MIS-C.

**Table 3:** Organ system involvement in hospitalized children and adolescents with MIS-C^*^.

**Organ system**	**Total cases (%)**	**MIS-C cases (%)**	**Non-MIS-C cases (%)**	***p*-Value**
	***N* = 41**	***N* = 18**	***N* = 23**	
Cases with more than 2 organ involvement	26 (63.4%)	18 (100%)	8 (34.8%)	<0.001
Gastrointestinal	17 (41.5%)	13 (72.2%)	4 (17.4%)	<0.001
Respiratory	16 (39%)	9 (50%)	7 (30.4%)	0.202
Hematologic	13 (31.7%)	12 (66.7%)	1 (4.3%)	<0.001
Dermatologic	10 (24.4%)	10 (55.6%)	(0%)	<0.001
Neurological	8 (19.5%)	6 (33.3%)	2 (8.7%)	0.048
Other (Rheumatological, Urogenital)	6 (14.6%)	5 (27.8%)	1 (4.3%)	0.035
Cardiac	3 (7.3%)	3 (16.7%)	(0%)	0.042
Renal	2 (4.9%)	2 (11.1%)	(0%)	0.101

*Multisystem Inflammatory Syndrome in Children with COVID-19, MIS-C.

**Table 4:** Investigations of children hospitalized with COVID-19, MIS-C^*^ vs. non-MIS-C.

Investigations requested**	**Total**	MIS-C*	**non-MIS-C**	***p*-Value**
**ESR (Normal** ** <10 mmhr)**	16 (39%)	14(77.8%)	2(8.7%)	
Mean	86	97.07	8.5	0.007
Standard Deviation, SD	47.089	38.72	6.36	
**CRP (Normal** **<** **1 mg/dL)**	18 (43.9%)	16 (88.9%)	2 (8.7%)	
Mean	11.4	12.2	5.1	0.512
SD	13.8	14.4	7.2	
**D-dimer (Normal** ** <500 ng/mL)**	17 (41.5%)	16 (88.9%)	1 (4.3%)	
Mean	6391.8	6711.6	1275	0.652
SD	11179.5	11465.5		
**Ferritin (Normal** **<** **315 ng/mL)**	13 (31.7%)	11 (61.1%)	2 (8.7%)	
Mean	715.5	765	443	0.634
SD	829	886.8	455.4	
**Lactate dehydrogenase (Normal** ** <190 U/L)**	20 (48.8%)	14 (77.8%)	6 (26.1%)	
Mean	519.5	558.9	427.7	0.443
SD	339.1	314.3	406.9	
**Neutrophil count (Normal** **<** **8)**	38 (92.7%)	18 (100)	20 (87%)	
Mean	8.7	11.2	6.5	0.024
SD	6.5	7.9	3.9	
**Lymphocyte count (Normal** **>** **1.5)**	38 (92.7%)	18 (100%)	20 (87%)	
Mean	2.5	1.9	3	0.056
SD	1.9	1.1	2.2	
**Albumin (Normal: 36–52 g/dL)**	23 (56.1%)	16 (88.9%)	7 (30.4%)	
Mean	34.2	33.8	35.1	0.706
SD	7.9	8.4	6.8	
**Electrocardiogram**	8 (19.5%)	8 (44.4%)	0 (0%)	
abnormal	2 (4.9%)	2 (11.1%)	0 (0%)	
**Echocardiogram**	14 (34.1%)	13 (72.2%)	1(4.3%)	
abnormal	3 (7.3%)	2 (11.1%)	1 (4.3%)	
**Abdominal Ultrasound**	4 (9.8%)	2 (11.1)	2(8.7%)	
abnormal	3 (7.3%)	2 (11.1%)	1(4.3%)	
**CT Chest**	2 (4.9%)	2 (11.1%)	0 (0%)	
abnormal	2 (4.9%)	2 (11.1%)	0 (0%)	
**CT Abdomen**	3 (7.3%)	1 (5.6%)	2 (8.7%)	
abnormal	1 (2.4%)	0 (0%)	1 (4.3%)	
**Lower Limb Doppler Ultrasound**	2 (4.9)	2 (11.1%)	0 (0%)	
abnormal	0 (0%)	0 (0%)	0 (0%)	
**Chest X-ray**	10 (24.4%)	6 (33.3%)	4 (17.4%)	
abnormal	6 (14.6%)	5 (27.8%)	1(4.3%)	

^*^Multisystem Inflammatory Syndrome in Children with COVID-19, MIS-C.

^**^ Fibrinogen, Interleukin 6, and Procalcitonin were not available.

Thirteen (72%) MIS-C cases had ≥4 abnormal inflammatory biomarkers, including D-dimers, C-reactive protein, ESR, ferritin, troponins, lactate dehydrogenase, neutrophils, platelets, lymphocytes, and albumen (Tables 4, 5). The MIS-C group's inflammatory markers were significantly more deranged than the non-MIS-C group, which included neutrophilia, anemia, and elevated ESR (Tables 4, 5). MIS-C cases had more abnormal radiological features, including pulmonary embolism, coronary arteritis, lung consolidation, pleural effusion, and pulmonary opacities than those presenting without MIS-C (Table 5). Thirteen (72.2%) echocardiograms were done in the MIS-C group, and 11.1% had abnormalities including coronary arteritis, mild mitral regurgitation, elevated pulmonary pressures, and a small pericardial effusion in a patient with Sickle cell disease (Table 4).

**Table 5:** Medical Intervention and outcomes of children hospitalized with COVID-19, MIS-C^*^ vs. non-MIS-C.

**Interventions and**	**Total cases (%)**	MIS-C*cases (%)	**Non-MIS-C cases (%)**	***p* Value**
**Outcomes**	***N* = 41**	***N* = 18**	***N* = 23**	
Intravenous Immunoglobulins (IVIg)	14 (34.1%)	14 (77.8%)	0 (0%)	<0.001
Anticoagulants	7 (17.1%)	5 (27.8%)	2 (8.7%)	0.209
Aspirin, ASA	12 (29.3%)	12 (66.7%)	0 (0%)	<0.001
Antiviral	2 (4.9%)	2 (11.1%)	0 (0%)	0.187
Broad Spectrum Antibiotics	28 (68.3%)	15 (83.3%)	13 (56.5%)	0.095
Corticosteroids	11 (26.8%)	9 (50%)	2 (8.7%)	0.005
**Complications**				
Anemia	3 (7.3%)	3 (16.7%)	0 (0%)	0.077
Pleural Effusion	3 (7.3%)	3 (16.7%)	0 (0%)	0.077
Pulmonary Embolism	1 (2.4%)	1 (5.6%)	0 (0%)	0.439
Cardiac Arrest	1 (2.4%)	1 (5.6%)	0 (0%)	0.439
Cardiac Arrhythmia	0 (0%)	0 (0%)	0 (0%)	-
Cardiomyopathy	0 (0%)	0 (0%)	0 (0%)	-
Coronary arteritis	1 (2.4%)	1(5.6%)	0 (0%)	0.439
Pneumonia	5 (12.2%)	3 (16.7%)	2 (8.7%)	0.638
Acute Respiratory Distress Syndrome, ARDS	2 (4.9%)	2 (11.1%)	0 (0%)	0.187
Duration of Hospitalization, days; Mean (SD)	11.41 (14.9)	12.4 (8.8)	10.55 (18.6)	0.693
Admission to ICU	2 (4.9%)	2 (11.1%)	0 (0%)	0.187
Transferred	1 (2.4%)	1 (5.6%)	0 (0%)	0.439
Discharged	39 (95.1%)	16 (88.9%)	23 (100%)	0.187
Demised	1 (2.4%)	1 (5.6%)	0 (0%)	0.439
Readmitted	2 (4.9%)	2 (11.1%)	0 (0%)	0.187
Readmitted — received Supportive Care	2 (4.9%)	2 (11.1%)	0 (0%)	0.187
Readmitted — received second IVIg and IV Steroids	1 (2.4%)	1 (5.6%)	0 (0%)	0.439
Readmitted — Discharge	2 (4.9%)	2 (11.1%)	0 (0%)	0.187
Readmitted — Demise	0 (0%)	0 (0%)	0 (0%)	-

MIS-C^*^-multisystem inflammatory syndrome of children with COVID-19.

Patients from both groups were treated for their presenting complaints (Table 5). Fourteen of the MIS-C group received intravenous immune gammaglobulin (77.8%), 12 (66.7%) received aspirin, and 9 (50%) high-dose corticosteroids. Other treatments included appendectomy in 2 (8.7%) non-MIS-C patients who presented with acute appendicitis. Antibiotics were administered in both MIS-C and non-MIS-C patients (Table 5).

Thirty-nine (95.1%) of 41 hospitalized children were discharged to follow-up. One (2.4%) patient with MIS-C commenced IVIgG, corticosteroids, and aspirin and was transferred to home country and successfully completed treatment. Readmissions occurred in three children. The first was a child with homozygous sickle cell disease (HbSS) who was hospitalized and treated for MIS-C with IVIgG, intravenous steroids, aspirin and improved. Two weeks later, she was readmitted with headaches, unilateral weakness of the lower limb, and worsening systemic inflammation requiring exchange blood transfusion, the second course of IVIgG, intravenous corticosteroids, and long-term anticoagulation, with the resolution of weakness by day 3. Another child with leukemia was readmitted ten days after initial hospital discharge and treatment for MIS-C, with altered mental status and delirium, which also resolved by day three. The third was a neonate who was born to a SARS-CoV-2 positive mother who was admitted with prolonged respiratory distress and small for gestational age. She was readmitted with significant neurodevelopmental challenges and autoimmune myasthenia gravis. There was one death (2.4%) from sudden myocardial dysfunction 13 days after testing SARS-CoV-2 RT-PCR positive, in a child who had Hemoglobin SC disease (HbSC) disease and was receiving hemodialysis for preexisting chronic kidney and bony metabolic disease.

## Discussion

This is the first formal report of COVID-19 in children from the Caribbean, in the multiethnic Jamaican subpopulation, describing the rate of admission, severity of illness, outcomes, and the differences between those children who had MIS-C and those who were acutely ill without MIS-C throughout various age ranges and comorbidities. These children presented in the first 15 months of the novel pandemic, when pediatric COVID-19 clinical epidemiology, case definitions, and treatment guidelines, were being defined globally, including here in Jamaica. Historically, it is important to track this disease in the Caribbean that is also an important subregion of the world. Our series is also different in that all hospitalized children were carefully identified and evaluated prospectively “case by case” for SARS-CoV-2 infection and MIS-C. The CDC published publicly available diagnostic criteria to identify MIS-C cases and also provided the resources to perform the relevant diagnostic investigations and administer early treatment with IVIgG. Training and active case surveillance may have contributed to identifying children and adolescents with a wider spectrum of diseases, including milder less severe cases, which were diagnosed and treated earlier in the illness with immune-modulating and anti-inflammatory agents, which probably aborted the inflammatory process earlier and likely contributed to our documented better outcomes.

This ambispective cohort study has also enabled improved clinical identification and development of best practices for SARS-CoV-2 infection in children and contributes to public health policy in Jamaica and treatment of children with SARS-CoV-2 infection and MIS-C from the Caribbean and similar developing countries. During the first 15 months of the pandemic, 79 children and adolescents who presented through UHWI's emergency room were later found to have evidence of SARS-CoV-2 infection and MIS-C. Forty-one of these 79 children were hospitalized, and all had written infectious diseases clinical consultations. Eighteen (44%) of the 41 hospitalized patients fulfilled criteria for MIS-C when the CDC's case definition was consistently and conscientiously applied. These data suggest that MIS-C may be more common than is recognized. Children from all age groups studied presented with SARS-CoV-2 infection. These cases occurred while SARS-CoV-2 infections in children, MIS-C, and the clinical and laboratory diagnostic criteria were being globally defined and as the treatment recommendations evolved. This case series captures children hospitalized during the COVID-19 pandemic in the first 15 months of its evolution in one regional academic medical center in a developing Caribbean island nation.

Overall, the patients did well, with or without MIS-C. They did not have fluid refractory shock or require inotropes, vasopressors, volume expanders, or invasive ventilation. Only 11.1% (2) MIS-C vs. 0% non-MIS-C patients required intensive care for noninvasive ventilation. This is significantly less than reported previously. In contrast, a meta-analysis of MIS-C patients revealed that 65.8% developed shock, 79.1% required admission to the intensive care unit (ICU), and 33.0% required mechanical ventilation (29). In Southern Turkey, Spain, and England, it was reported that 17.3, 84.4, and 50% of children with MIS-C presented with shock, respectively; while 28.8, 13.3, and 79% required ICU admission (30–32). In the USA, 51.0% received vasopressors and 15.0% mechanical ventilation in New York, and a national surveillance study in the US of children with MIS-C reported 45.1% had shock and required vasopressors and 9.4% required invasive ventilation over the first three waves of the pandemic (33, 34). While a pediatric case series in Qatar reported 71.4% requiring inotropes while in intensive care (35). In the developing world, reports from Mexico and Pakistan reported 23 and 25% of children with MIS-C in shock, respectively (36, 37).

The significantly improved outcomes that were observed in our study may be multifactorial. There was increased surveillance with a high index of suspicion by clinicians, widespread educational sessions on COVID-19 and MIS-C, early and throughout the pandemic. This led to increased knowledge and application of the CDC's MIS-C case definition which were simple to interpret and implement. Case surveillance and pediatric infectious disease consultations led to early referrals to and within our regional academic hospital, prompt diagnosis and aggressive treatment of MIS-C with IV immunoglobulin, steroids, and aspirin, as well as monitoring of inflammatory markers, which likely led to more favorable outcomes with successful hospital discharge rates for patients with MIS-C (94.4%) and without MIS-C (100%). In our study, IV immunoglobulin was given to 77.8% of our MIS-C patients and 50% also received IV glucocorticoids. This may be beneficial in resource-constrained settings, such as Jamaica. However, IV immunoglobulin is costly and may affect accessibility and outcomes in children as the pandemic progresses. A study from the United States reported initial treatment with IV immunoglobulin and IV glucocorticoids at diagnosis of MIS-C led to reduced left ventricular dysfunction and need for vasopressors than if IV immunoglobulin was administered alone (38). Measures must be implemented to ensure that all children have equal access to these lifesaving medications once MIS-C is diagnosed. WHO has since recommended steroids for the treatment of MIS-C in developing countries and other resource-constrained settings, where IV immunoglobulin may be unavailable.

The outcomes in our study may be linked primarily to the B.1.1.7 (Alpha) SARS-CoV-2 “variant of concern,” the ancestral strain, and other minor variants, which were circulating in Jamaica during the period of our study (24–26). This may be equated to the experiences in other countries, although most reported more severe MIS-C outcomes during the same 15-month period of the global pandemic.

The majority of our patients were “Afro-Caribbean.” The ethnically diverse nature of being “Caribbean” may also be protective. A prospective cohort study in the US reported “Black” and “Hispanic” race as predictors for hospitalization in children with COVID-19; 19.5% of hospitalized children required intensive care (39). Apolipoprotein E4 (apoE4) occurs high in frequency in individuals of African descent as compared to Europeans or Asians and was considered to be a predictor of rapid and severe illness in COVID-19, as it leads to robust immune responses (40, 41). Jamaica, a Caribbean Island, is considered ethnically-diverse (National Motto – ‘Out of Many, One People') because of the many populations that initially settled in the region, including mixes of European, African, Asian, Indian, Indonesian Javanese, Chinese, and Aboriginal Indians (42).

Children with MIS-C had a wide array of organ-system involvement and abnormal inflammatory markers. Both simple, more accessible inflammatory markers, such as complete blood count, lactate dehydrogenase, ESR, and CRP, as well as the more costly and less accessible markers in the developing world, such as D-dimers, ferritin, and procalcitonin, were made available to our patients and were abnormal. These are all characteristic of the multisystemic inflammatory nature of MIS-C (41–48). Just under a quarter of the MIS-C cases were SARS-CoV-2 RT-PCR positive and of the RT-PCR negative MIS-C cases, 44.4% had positive IgG/IgM antibodies and the remaining 33.3% had confirmed, or presumed contact usually with infected household family members, especially during the periods of widespread and intense community transmission. This is congruent with the post-infectious nature of MIS-C, which develops up to six weeks or more after initial exposure to SARS-CoV-2. Many institutions in Jamaica do not have access to SARS-CoV-2 antibody testing, despite its availability in our academic center. The lack of testing could lead to evidence of SARS-CoV-2 being missed and families' unawareness of their child's initial exposure, or acute infection, which may be asymptomatic. Despite these challenges, conscientious application of the CDC's case definition during intense community transmission, assisted clinical diagnosis and implementing treatment promptly, since community transmission increases the likelihood of SARS-CoV-2 exposure. Antibody testing and high index of clinical suspicion also improved MIS-C diagnosis and facilitated treatment. A study from the USA reported that MIS-C followed their COVID-19 community peaks in two to five weeks (49). All patients in our study who were identified clinically as MIS-C without serological evidence (33%), demonstrated multisystem involvement and abnormal inflammatory markers, without an alternate etiology to explain their symptoms. All children treated for MIS-C had improved clinical findings and inflammatory markers once therapy was started.

Cardiac decompensation, though present in under one-fifth of our sample population, was not a significant feature in our study, in contradistinction to other published reports (29, 31, 39, 41–46, 48). Our findings may be attributed to a disease that was not very severe, associated with prompt diagnosis and treatment. The variant alpha strain or the ethnicity may also be significant (23, 24, 41, 42). Challenges in accessing echocardiograms and/or electrocardiograms in the acute setting in our developing country may have also led to radiological under-representation. However, our patients did not have clinical cardiac decompensation, and troponins, when performed, were not frequently abnormal. One patient, with multiple comorbidities including Hemoglobin SC disease and chronic renal failure requiring hemodialysis, had sudden cardiac dysfunction and died 13 days after his positive SARS-CoV-2 RT-PCR result. The prevalence of comorbidities was similar in our MIS-C and non MIS-C patients. Globally, comorbidities have been noted to lead to more severe outcomes (30–33, 36–39, 49, 50). Children and adolescents should therefore be prioritized for COVID-19 vaccinations within their age bands, once authorized and accessible. However, COVID-19 vaccines may be challenging to access for pediatric cases in developing countries and the elderly and those with comorbidities are at higher risk for death from COVID-19 and are usually prioritized ahead of children (20).

Laboratory confirmation of SARS-CoV-2 infection was not uniformly available throughout the study for a small minority of the patients, as the global pandemic evolved, laboratory investigations were being refined and community exposures were uniformly accepted in all of the diagnostic criteria (8–12). MIS-C, a post-inflammatory response, occurs up to six weeks or more after the initial SARS-CoV-2 exposure, which may be associated with an initial symptomatic or asymptomatic infection. Other investigations were not readily available acutely, in our developing country setting. However, 72% of patients with MIS-C obtained echocardiograms later and few had cardiac involvement. A few patients did not return or respond to telemedicine follow-up visits to determine post-hospital ambulatory outcomes.

In conclusion, of 79 children and adolescents who presented with SARS-CoV-2 infection and/or MIS-C to UHWI, the regional academic Medical Center in Jamaica during the first 15 months of the global pandemic, 52% were hospitalized and of these 44% were diagnosed with MIS-C when the CDC's case definition was uniformly and consistently applied. MIS-C seemed more common than is recognized. Non-MIS-C patients were mild and were treated successfully with standard care. In symptomatic non-MIS-C patients, it was often challenging to ascribe overlapping clinical features to the comorbidities or to their acute SARS-CoV-2 infection (e.g., Sickle cell disease, asthma, appendicitis). Ninety-four percent of MIS-C patients were successfully treated promptly with IV gamma globulin, steroids, and/or aspirin most likely expediting their recovery before cardiopulmonary decompensation and the need for inotropes, vasopressors, or invasive ventilation. One death from myocardial dysfunction occurred in a child with premorbid homozygous sickle cell disease and chronic renal failure who was receiving renal replacement therapy. While MIS-C cases were not very severe in this series, the high index of suspicion, widespread training (51–54), active surveillance, and correct application of the CDC's MIS-C diagnostic criteria, with prompt diagnosis and treatment likely contributed to early diagnosis of more cases and improved outcomes in children in this resource-constrained multi-ethnic Caribbean developing country.

## Author's note

An abstract was published, and a poster presentation of this manuscript has been displayed and was virtually presented by Crista-Lee Shahine Berry to the American Academy of Pediatrics (AAP), National Conference and Exhibition, on 8–12 October 2021 in the Section on “Global Health Program” (VH1309), at the Philadelphia Convention Center, Pennsylvania, USA. The paper was also uploaded to the preprint server on 26 November, 2021 *viz*: Berry C, Melbourne-Chambers R, Harrison A. Anzinger J. Gordon Johnson K., Deyde V, Christie CDC. “COVID-19 Clinical Characteristics and Outcomes in Children Aged <16 years at the University Hospital of the West Indies, Jamaica in 2020-2021.” Preprint–medRxiv 2021.11.26.21266916; doi: 10.1101/2021.11.26.21266916.

## Data availability statement

The raw data supporting the conclusions of this article will be made available by the authors, without undue reservation.

## Ethics statement

The studies involving human participants were reviewed and approved by the Mona Campus Research Ethics Committee of the University of the West Indies and protocol clearance by the Division of Global HIV and Tuberculosis (DGHT) Science Integrity Branch of the United States Centers for Disease Control and Prevention (CDC). Clinical data were collected from clinical records only (and in the context of an epidemic), hence there was exemption for consent/assent from patients, parents and guardians. Written informed consent from the participants' legal guardian/next of kin was not required to participate in this study in accordance with the national legislation and the institutional requirements.

## Author contributions

C-LB, RM-C, AH, JA, and CC acquired the data. C-LB, K-AG-J, and VD performed data analysis, and everyone commented on the proposal, the work and data interpretation throughout. C-LB and CC concepted, designed the work, drafted and revised the work. All authors contributed to the process throughout and approved the final version submitted for publication.

## Funding

The project described was supported by the Centers for Disease Control and Prevention CDC under the terms of project number: CDC#19JM3710P1001.

## Conflict of interest

The authors declare that the research was conducted in the absence of any commercial or financial relationships that could be construed as a potential conflict of interest.

## Publisher's note

All claims expressed in this article are solely those of the authors and do not necessarily represent those of their affiliated organizations, or those of the publisher, the editors and the reviewers. Any product that may be evaluated in this article, or claim that may be made by its manufacturer, is not guaranteed or endorsed by the publisher.

## Author disclaimer

The findings and conclusions in this report are those of the author(s) and do not necessarily represent the official position of the funding agencies.
